# Editorial: Microbial Degradation of Plastics

**DOI:** 10.3389/fmicb.2021.635621

**Published:** 2021-02-11

**Authors:** Ren Wei, Nick Wierckx

**Affiliations:** ^1^Junior Research Group Plastic Biodegradation, Institute of Biochemistry, University of Greifswald, Greifswald, Germany; ^2^Institute of Bio- and Geosciences IBG-1: Biotechnology, Forschungszentrum Jülich GmbH, Jülich, Germany

**Keywords:** plastic pollution, plastic degradation, microorganisms, recycling, upcycling

The accumulation of mismanaged plastic waste in natural environments poses a serious threat to our oceans, wildlife, and human health. As the global demand on plastics is growing continuously, the trend of plastic emission into open environments is unlikely to reduce until 2030 unless key policies, consumption behaviors and waste management measures regarding plastic products will be radically transformed immediately (Borrelle et al., 2020).

While abiotic environmental degradation contributes considerably to the fragmentation of large plastic debris resulting in micro- and nanoplastic pollution (Min et al., 2020), the role of microorganisms in the plastic degradation under natural conditions is still poorly understood. In recent years, various microbes have been reported capable of depolymerizing synthetic polymers at laboratory conditions (Wierckx et al., 2018). Nonetheless, the microbial degradation extents and rates of conventional petroleum-based plastics such as polyethylene (PE) and polystyrene (PS) can differ remarkably to those of biodegradable polyesters such polylactic acids (PLA). Microbial biotechnology has been repeatedly proposed as an option of sustainable disposal approach of plastic waste although the reality and promise of biotechnological recycling methods is not yet unambiguously clarified among scientific communities, plastic end-users and policy makers (Wei et al., 2020).

As a response to this research field of rapidly increasing interest, we proposed this Research Topic and could finally collect 11 high-quality contributions based on both original research and literature survey by scientists from all over the world.

Ru et al. provided a comprehensive review on the microorganisms and enzymes able to degrade mass-produced recalcitrant petrochemical plastics reported since the 1970s. While PE and PS have been shown to be degraded by several microorganisms, albeit very slowly, the key depolymerases involved in the breakdown of carbon-carbon backbones remain still unknown. For a better understanding of the enzymatic degradation of vinyl polymers, Xu et al. carried out quantum mechanism calculations to model the cleavage of the carbon-carbon bond at the C position under both acidic and alkaline conditions.

Microbial communities are a valuable source of enzymes with degrading activities on synthetic polymers. Pinnell and Turner reported the shotgun metagenomic sequencing of biofilms fouling polyethylene terephthalate (PET), polyhydroxyalkanoate (PHA) and ceramic placed at the sediment-water interface of a coastal lagoon. While PET plastic biofilms were indistinguishable compared to the ceramic biofilm control, PHA bioplastic biofilms were distinct as indicated by the dominant presence of sulfate-reducing microorganisms (SRM) and a significant enrichment of phylogenetically diverse polyhydroxybutyrate (PHB) depolymerases. Their findings indicate that the plastisphere SRM play an important role in PHA biodegradation. Gaytán et al. studied the polyurethane (PUR) degrading activity of a landfill microbial mixed culture which can grow on aqueous PUR dispersion as a sole carbon source. Physiochemical analyses suggested the cleavage of various functional groups in the polymers. Along with the metagenomic analysis, tentative microbial PUR degradation pathways were proposed. Weinberger et al. cultivated a fungal library with aliphatic and aromatic polyesters, leading to the identification of new strains that produce polyester hydrolyzing enzymes. This method will enable exploring the available fungal diversity and thus broadening the spectrum of candidate enzymes for plastic recycling. The increasing commercial demand on bio-based and biodegradable plastics such as PLA requires also environment-friendly disposal methods using microorganisms (Butbunchu and Pathom-Aree). By summarizing the findings about PLA degradation by Actinobacteria since 1997, *Pseudonocardiaceae* are identified as the most important family. Moreover, esterases and proteases found in various Actinobacteria capable of degrading PLA will hold the promise for future application in the bioplastic waste management.

Many studies have focused on the enzymatic degradation of PET during the last decade (Wei and Zimmermann, 2017). Bollinger et al. reported the high-resolution structural elucidation and functional characterization of a novel PET hydrolyzing enzyme (PE-H) identified in the genome of the marine bacterium *Pseudomonas aestusnigri*. PE-H was shown to degrade amorphous PET at 30°C. By structural modeling and mutagenesis study, a Y250S variant was constructed to exhibit increased PET hydrolytic activity as a result of the rearrangement of the active site conformation, thereby providing new knowledge on the structural features required for efficient polyester degradation. Based on previous reports regarding the PET polymer chain mobility associated with enzymatic degradation (Wei et al., 2019a,b), Falkenstein et al. could evaluate the feasibility of UV radiation as a potential pretreatment method to stimulate the subsequent biocatalytic depolymerization of PET. Although UV treatment has caused significant chain scissions at the surface layer of amorphous PET films, the resulting increased surface crystallinity drastically impaired the efficiency of enzymatic degradation.

As engineered whole-cell catalysts have been recently considered with great potential for plastic degradation (Yan et al., 2020), the microbial metabolism of plastic monomers and additives will become a research focus both in the contexts of environmental degradation of plastic pollution and of biotechnological plastic upcycling, i.e., utilization of plastic hydrolysates as feedstocks for microbial production of chemicals with high value (Wierckx et al., 2015; Salvador et al., 2019; Ru et al.). Carstens et al. characterized the biotransformation of phthalate esters, ubiquitously used as plasticizers, by marine, freshwater, and terrestrial fungi. This study indicated an important role of fungi in the environmental biodegradation of complex plastic-derived molecules. Cárdenas Espinosa et al. isolated *Pseudomonmas* sp. TDA1 which was able to grow on an oligomeric PUR diol solution, as well as on the PUR-derived 2,4-diaminotoluene. Li et al. took a laboratory evolution and reverse engineering approach to enable efficient metabolism of 1,4-butanediol by *P. putida* KT2440, and to characterize the underlying pathways and their regulation. This work builds upon the previous engineering (Franden et al., 2018) and evolution (Li et al., 2019) of *P. putida* KT2440 on ethylene glycol. One third of the original research papers are focused on Pseudomonads, including two on monomer metabolism (Cárdenas Espinosa et al.; Li et al.) and one as a source of plastic-degrading enzymes (Bollinger et al.). This is not surprising given the conspicuous potential of this organism in this field of research (Wierckx et al., 2015; Wilkes and Aristilde, 2017; Schwanemann et al., 2020; Ru et al.), especially concerning plastics upcycling (Tiso et al., 2020).

In summary, this Research Topic collects 11 scientific contributions which consolidate and expand our knowledge on the recent advances in this very active research field. We sincerely hope that more scientists will be inspired and encouraged by our Research Topic to make their own contributions to tackle the global challenge caused by plastic pollution.

## Author Contributions

All authors contributed to writing and approval of this editorial.

## Conflict of Interest

The authors declare that the research was conducted in the absence of any commercial or financial relationships that could be construed as a potential conflict of interest.
